# Xenoimplant of Collagen Matrix Scaffold in Liver Tissue as a Niche for Liver Cells

**DOI:** 10.3389/fmed.2022.808191

**Published:** 2022-04-07

**Authors:** Moises Martinez-Castillo, Benjamín León-Mancilla, Gerardo Ramírez-Rico, Ana Alfaro, Armando Pérez-Torres, Daniela Díaz-Infante, Jorge García-Loya, Zaira Medina-Avila, Jaime Sanchez-Hernandez, Cristina Piña-Barba, Gabriela Gutierrez-Reyes

**Affiliations:** ^1^Liver, Pancreas and Motility Laboratory, Unit of Research in Experimental Medicine, School of Medicine, Universidad Nacional Autónoma de México (UNAM), Mexico City, Mexico; ^2^Facultad de Estudios Superiores Cuautitlán, Universidad Nacional Autónoma de México (UNAM), Cuautitlán Izcalli, Mexico; ^3^Department of Pathology, Hospital General de México, Mexico City, Mexico; ^4^Department of Cells and Tissue Biology, School of Medicine, Universidad Nacional Autónoma de México (UNAM), Mexico City, Mexico; ^5^Materials Research Institute, Universidad Nacional Autónoma de México (UNAM), Mexico City, Mexico

**Keywords:** liver, collagen matrix scaffold, xenoimplant, animal model, cell niche

## Abstract

Hepatitis C virus-induced liver damage, chronic liver damage due to alcohol, and non-alcoholic liver disease-induced cellular alterations promote fibrosis, cirrhosis, and/or hepatocellular carcinoma. The recommended therapeutic option for advanced liver damage is liver transplantation. Extracellular matrix scaffolds have been evaluated as an alternative for tissue restoration. Studies on the biocompatibility and rejection of synthetic and natural scaffolds as an alternative to organ transplantation have been evaluated. Our group has recently described the xenoimplant of collagen matrix scaffold (CMS) in a rat model. However, no complete macroscopic and histological description of the liver parenchyma at the initial (day 3), intermediate (day 14), and advanced (day 21) stages has been obtained. In this study, we described and compared liver tissue from the CMS zone (CZ, CMS, and liver parenchyma), liver tissue from the normal zone (liver parenchyma close to the CMS), and basal tissue (resected tissue from the CMS implantation site). Our data strongly suggest that the collagen matrix xenoimplant is a good niche for hepatocytes, with no rejection, and does not affect liver function tests. The liver can regenerate after damage, but this capacity is inhibited in a chronic injury. At present, the use of CMS after liver damage has not been reported. This biomaterial could be a novel alternative in the field of regenerative medicine for liver diseases.

## Introduction

Liver disease causes approximately, 2 million deaths per year worldwide. Cirrhosis is one of the most common complications, which can be induced by viruses, alcohol abuse, and non-alcoholic fatty liver disease. Some of these factors promote liver failure and progression to hepatocellular carcinoma (1, 2). Liver transplantation is the second-highest ranked solid organ transplant. However, less than 10% are successfully carried out (3). Biocompatibility, low donation rates, preoperative and postoperative management, high costs, and ethical considerations, among other items, play an important role in organ transplantation (4–6). In this context, regenerative medicine has demonstrated that biomaterials (scaffolds) are an excellent option for avoiding the limitations and difficulties of organ transplantation (7, 8).

Collagen has been reported to be the most abundant protein found mainly in the stroma of organs (9). Over the past decade, our group has been evaluating the biochemical and physical properties of natural Nukbone**^®^**, which is obtained from bovine animals (10). This biomaterial is composed of hydroxyapatite and collagen I and has shown satisfactory results in the field of odontology, orthopedics, maxillofacial surgery, and plastic and reconstructive surgery (11). We recently reported that Nukbone^®^, when treated with chloride acid, enables the polymeric biomaterial to be maintained with low hydroxyapatite (10–15%) while preserving collagen I and its porous structure (12). This biomaterial was named collagen matrix scaffold (CMS). The preclinical evaluation of CMS showed it to be a well absorbable bioprosthesis. Bile duct injury was induced in a porcine model, followed by choledochectomy and CMS implantation. Biosorption of the material occurred 6 months after the surgical procedure, with no tissue alteration or evidence of stenosis (13). In addition, we recently reported the implantation of CMS in the livers of rats. However, the full histological events and characterization of the model were not reported (14).

It is well known that the liver is a very efficient regenerative organ. In experimental animals, excision of up to two-thirds of the liver from a healthy animal did not promote signs of hepatic dysfunction, and the liver mass was rapidly compensated by hyperplasia (15). The regeneration process of hepatocytes also includes the replication of bile duct epithelium, endothelium, and sinusoidal lining cells, which leads to an increase in the size of the existing lobules. However, some studies suggest that lobule formation is due to the subdivision of existing lobules. When hepatocyte reproduction is inhibited in chronic or severe disease, the multiplication and differentiation of liver cells are orchestrated by stem cells and progenitor cells (16, 17). Progenitor cells reside in the cholangiole (the canal of Hering). During proliferation, these cells can be organized in islands or in immature tubules of small basophilic cells; this process is known as ductular reaction (18, 19). An important consideration is the fact that, during chronic liver disease, the intense production of extracellular matrix (ECM) proteins in fibrosis stages promotes an allosteric effect, reducing the space for the proliferation of hepatocytes and other parenchymal cells. Thus, the scaffold abrogates this negative effect of excessive ECM production. In this study, we provided histological evidence at days 3, 14, and 21 after the implantation of CMS in liver tissue. The CMS replaced the extirpated liver mass (40%), and no evidence of xenoimplant rejection was observed at day 21 of evolution. This biomaterial did not display rejection or organ dysfunction. The implantation of natural scaffolds in hepatic diseases could provide a good niche for the formation of cell nodules with a similar phenotype to normal liver parenchyma.

## Materials and Methods

### Obtention of Collagen Matrix Scaffold

To obtain the CMS, we collected the bovine femoral condyle, as previously reported (14). Briefly, the selected samples of 3 cm × 3 cm were carefully dissected and washed with water using anionic detergent. We then cut a triangular piece that was 1 cm on each side and 0.4–0.5 cm thick (Nukbone)^®^. Posteriorly, the biomaterial was demineralized with 0.5 M HCl (Merck, Millipore, United States) for 10 min and washed with distilled water to obtain the CMS (12), which was then sterilized using the hydrogen peroxide vapor/plasma sterilization method (20). The biomaterial was provided by the Materials Research Institute, *Universidad Nacional Autónoma de México* (*UNAM*).

### Animal Model

Wistar male rats, weighing 250–300 g, were provided by the Laboratory Animal Facility of the School of Medicine, *UNAM*. The animals were subdivided into three groups (*n* = 5 per group): (1) partial hepatectomy (PH) with no CMS implantation, (2) PH plus CMS implantation (PH + CMS), and (3) animals with no hepatectomy (sham group). The liver parenchyma of the animals in each group was evaluated at the initial (day 3), intermediate (day 14), and advanced (day 21) stages.

This study was approved by the Ethics Committee of the School of Medicine at the *UNAM*. All procedures were performed according to official Mexican policy (21). Our institution fulfills all technical specifications for the production, care, and use of laboratory animals and is certified by national law (*NOM*-062-ZOO-1999).

### Hepatectomy and Collagen Matrix Scaffold Implantation

Partial hepatectomy (40% of the left lobe) and CMS implantation were performed, as previously described (14). Briefly, the animals were sedated using intramuscular doses of ketamine (35 mg/kg) and xylazine (2.5 mg/kg) (*PiSA*, *Agropecuaria*, Mex). The abdominal surface was shaved and cleaned using chlorhexidine. A midline incision was made using a scalpel, after which the left lobe of the liver was exposed and placed on a metal plate. Using a metallic guide, 40% of the liver tissue was then removed. For the sham group, after its exposure, the liver was returned to the abdominal cavity and the animals were sutured. In the PH group, once hemostasis was achieved after excising the liver fragment (basal tissue), the animals were sutured. Regarding CMS implantation, the biomaterial was implanted in the extracted area, using four stitches of non-absorbable polypropylene suture (7-0 Atramat^®^, Mex). In all the cases, the muscle and skin were sutured using a 3-0 Dermalon suture (Medtronic MITG-Covidien, United States).

### Postoperative Care and Histopathologic Processing

After the surgical procedure, the rats were placed on a heating mattress, and 2.5 mg/kg of flunixin meglumine (*PiSA*, *Agropecuaria*, Mex) was administered to ameliorate pain. Each rat was housed in an individual polycarbonate box, and water and food were provided *ad libitum*. At the end of each evaluation time (days 3, 14, and 21), euthanasia was carried out using an intraperitoneal overdose of sodium pentobarbital (*PiSA*, *Agropecuaria*, Mex).

Exploratory laparoscopy was performed on all the animals in each group, and the liver tissue samples from the normal and CMS zones (CZs) were selected and fixed with 4% formalin for 24 h. The liver samples were dehydrated with gradual concentrations of alcohol (60, 70, 80, 90, and 100%) and embedded in paraffin, after which 4 μm semi-fine sections were cut from the paraffin blocks for hematoxylin and eosin (H&E) (Sigma Aldrich, United States) and Masson’s trichrome (Sigma Aldrich, United States) staining. The histopathologic processing was carried out by one histotechnologist, and the histological analysis was performed by three expert histopathologists; two of them with expertise in human samples and one with experience in animal liver samples. Images were observed using light microscopy (Nikon, Japan). All of the samples were evaluated, and the representative areas were selected. The micrographs were obtained at 4 ×, 10 ×, and 40 × magnifications, and the data were processed using Nikon ACT-1 software.

### Biochemical Evaluation of Liver Function

Before the *albus line* incision (basal values), a total of 500 μl of blood was drawn from the lateral tail vein to evaluate the liver function tests and explore other biochemical parameters. A blood sample from the sham group and the animals with CMS implantation was also drawn at the end of each time point (days 3, 14, and 21). Samples were centrifuged at 3,000 rpm/10 min, and the serum obtained was stored at −80°C until use. The biochemical parameters included albumin (ALB), bilirubin (BIL) (total and direct), cholesterol (CHOL), triglycerides (TG), glucose (GLU), uric acid (UR ac), creatinine (CR-S), blood urea nitrogen (BUN), alkaline phosphatase (ALP), alanine aminotransferase (ALT), and aspartate aminotransferase (AST).

### Statistical Analysis

The biochemical data were obtained from three animals at each time point and condition, and the values were reported as mean ± standard deviation (SD). The intragroup and intergroup comparisons were performed using the one-way ANOVA analysis and the Tukey-Kramer *post hoc* test. Data were expressed as mean ± standard error. A *p*-value of < 0.05 was considered statistically significant. Statistical analyses were performed using GraphPad Prism version 8.0.0 for Windows (GraphPad Software, San Diego, CA, United States)^1^.

## Results

### Collagen Matrix Scaffold Implantation Substituting the Removed Liver Fragment

**FIGURE 1:**
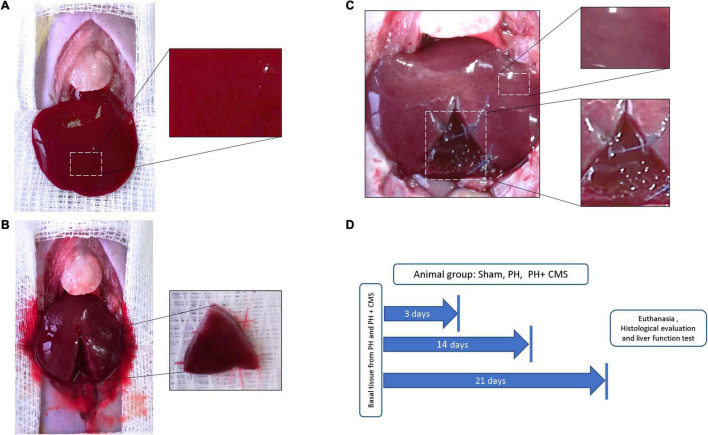
Animal groups. **(A)** Rats without hepatectomy (sham). Right inset, normal hepatic parenchyma. **(B)** Rats with partial hepatectomy (PH). Triangular dissected basal tissue (inset). **(C)** Rats with PH + CMS. Normal liver zone (upper inset), regeneration area where the collagen matrix scaffold (CMS) was implanted (lower inset). **(D)** Timeline with the experimental design.

To evaluate the CMS xenoimplant as a natural scaffold of liver cells, a 40% hepatectomy plus CMS was performed on the animal model (Figure 1C). Liver samples from the sham, 40% PH, and PH + CMS groups were compared (Figures 1A–C). Overall, liver samples from the basal fragment and sham animals had a slightly friable consistency and were reddish-brown (Figures 1A,B). The PH + CMS samples were white, mainly in the intermediate zone of the lobe, accentuating the liver lobules. The limits between the CMS and native liver tissue were considered the transition zone, whereas the CZ involves the tissue present in the CMS (Figure 1C, inferior inset), and the normal zone (NZ) was the native liver tissue 2 cm from the implantation site (Figure 1C, superior inset). The time points in the experimental design were days 3, 14, and 21, and euthanasia was carried out on those days as well (Figure 1D).

### Macroscopic Evaluation of the Animals

**FIGURE 2:**
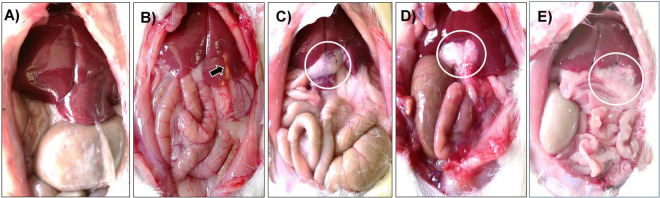
Macroscopic evaluation of animals after surgical procedures. Representative laparoscopy examination of the sham **(A)** and PH **(B)** animals at day 21; analysis showed normal organs. The PH group showed a scar at the 40% hepatectomy site (arrow). The PH + CMS group at days 3 **(C)**, 14 **(D)**, and 21 **(E)** showed evident adipose tissue surrounding the xenoimplant (circles) but with no adjacent organ alteration or infection.

The animals were observed after the surgical procedure. The survival rate for the animals in the sham and PH groups was 95%, whereas the survival rate for the animals in the PH + CMS group was 90, 85, and 90% at days 3, 14, and 21, respectively. As expected, the sham group animals displayed no changes in the abdominal cavity, and all the organs showed normal position, size, and shape, even at day 21 (Figure 2A). The animals that underwent PH showed adipose tissue from the epiploon, with no evidence of infection or abdominal organ alteration. Discrete cicatricial tissue was observed on day 21 (Figure 2B). In the PH + CMS group, adipose tissue was observed around the border of the CMS and native tissue at post-implantation days 3, 14, and 21. Moreover, the other liver lobes were normal in color and texture and showed no signs of infection or organ alteration (Figures 2C–E). To resect the samples from the CZ, the adipose tissue covering the implanted biomaterial had to be removed. On day 21, the biomaterial showed macroscopic evidence of reabsorption that correlated with our previous report. Importantly, the animals displayed no changes in conduct, and the intake of food and water was normal in all the groups. Furthermore, there were no signs of infection around the sutures in any of the animals. The lack of any local or systemic alterations in the macroscopic findings suggests that the xenogenic implant was not rejected.

### Incipient Inflammation in the Collagen Matrix Scaffold, but Not in the Native Liver Parenchyma, at Post-implantation Day 3

To understand and describe the histological changes in each group, the samples were randomly supplied to pathologists who had no internal or external influence on the study. A total of ten independent areas were described and consolidated.

**FIGURE 3:**
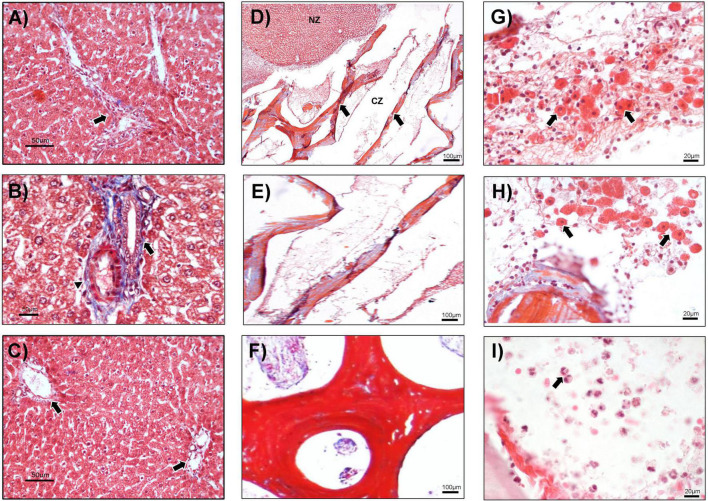
Histological analysis at day 3. **(A)** The liver without hepatectomy (sham) with triad portal (arrow), 100×. **(B)** In the PH group, the biliary duct (arrow) and hepatic artery (arrowhead) are shown, 400×. **(C)** Basal tissue from the animals at implantation day 3, in which the typical arrangement of liver parenchyma and portal triads are observed (arrows), 100×. **(D–I)** PH + CMS animals. **(D)** Normal zone (NZ) and CMS zone (CZ), liver tissue with CMS (arrows), 40×. **(E)** CMS zone (RZ) at high magnification, 100×. **(F)** Trabeculae and pore of CMS, 400×. **(G,H)** Hepatocyte-like cells (arrows) around the CMS, 100×. **(I)** Inflammatory cells (arrows) in the CMS, 100×. Representative images with Masson’s trichrome staining.

After 3 days of study, the sham group, basal sample, and NZ displayed the typical distribution of the liver parenchyma (Figures 3A–C). Open-faced (predominantly euchromatin) hepatocyte cords, some of which had double nuclei, were observed. Granular cytoplasm that could have resulted from an abundance of endoplasmic reticulum and mitochondria was seen (Figures 3A–C). Normal-diameter blood vessels that contained a lower number of erythrocytes were also observed. However, intense distribution of Küpffer cells in the sinusoids and portal triads was detected (Figures 3A,C).

In contrast, the CZ showed areas of hepatocytes with normal morphology and thick collagen bands from the CMS (Figures 3D,F). Interestingly, no evidence of inflammatory reaction was observed in the native parenchyma or at the border of the CMS with the native tissue (Figures 3D,E). Moreover, there was a discrete presence of hepatocyte-like cells surrounded by mononuclear cells in stromal tissue around the CMS. These observations suggest the possible migration of cells from the normal parenchyma area, or NZ, that did not yet represent a regeneration process (Figures 3G,H). A slight inflammatory reaction composed of neutrophils was also noted (Figure 3I).

### The Collagen Matrix Scaffold Xenoimplant Promotes the Formation of Cell Nodules, Fibroplasia, and Angiogenesis in Liver Tissue

**FIGURE 4:**
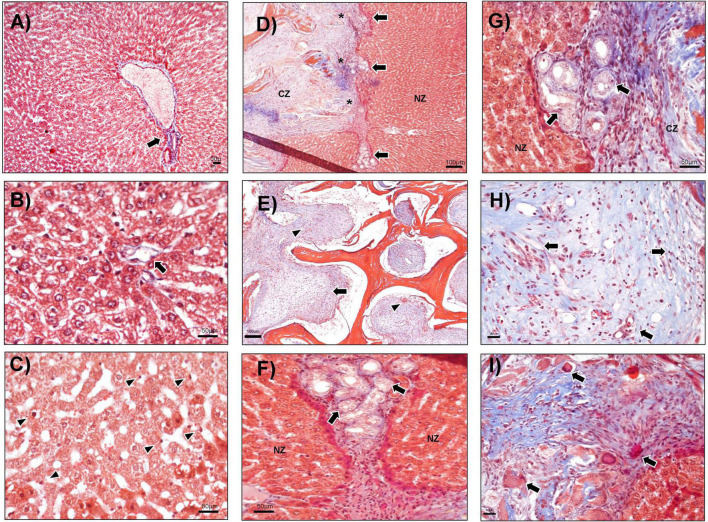
Histological events at day 14 of evolution. **(A)** Representative sham animals showed portal triads (arrow) and normal parenchyma, 100×. **(B)** PH group: central vein is shown (arrow), 400×. **(C)** Basal fragments from animals at day 14 displayed the normal distribution of hepatocyte cords and Küpffer cells (arrowhead), 400×. **(D–I)** PH + CMS. **(D)** CMS zone (CZ), ductular reaction shown (arrows), and multifocal areas of inflammation (asterisks). Normal zone (NZ) showed a typical parenchyma arrangement, 40×. **(E)** The CZ showed CMS with great neovascularization (arrowheads) and fibroplasia (arrow) between CMS, 40×. **(F,G)** At the limits of the CZ, a ductular reaction is observed in both the NZ and CZ (arrows), 400×. **(H)** CZ with neovascularization (arrows) between the CMS, 400×. **(I)** Areas of granulomatous inflammation with giant cells (arrows). Representative images with Masson’s trichrome staining.

On day 14, the sham group animals displayed the typical distribution of the hepatic parenchyma, the same as the basal tissue (Figures 4A–C). In contrast, a transition area between native tissue and the CMS was observed in the CZ. In the sham group, hepatocytes with normal morphology and multiple portal triads were predominant (Figure 4D), whereas abundant proliferation and infiltration of connective tissue (fibroplasia) were found in the CZ (Figure 4E), as well as large areas of neovascularization (angiogenesis) (Figure 4H), corresponding to granulation tissue. A ductular reaction characterized by undefined small-caliber ducts with lumen was formed in both the CZ and NZ (Figures 4F,G). A ductular reaction usually indicates the intense proliferation of progenitor cells. Focal inflammation of mononuclear cells, including lymphocytes, macrophages, Langhans-type, and foreign-body giant cells (FBGCs; granulomatous inflammation), was reported at the boundaries between the liver tissue and CMS (Figure 4I).

### Liver Parenchyma Without Inflammation or Rejection of the Xenoimplant at Postimplantation Day 21

**FIGURE 5:**
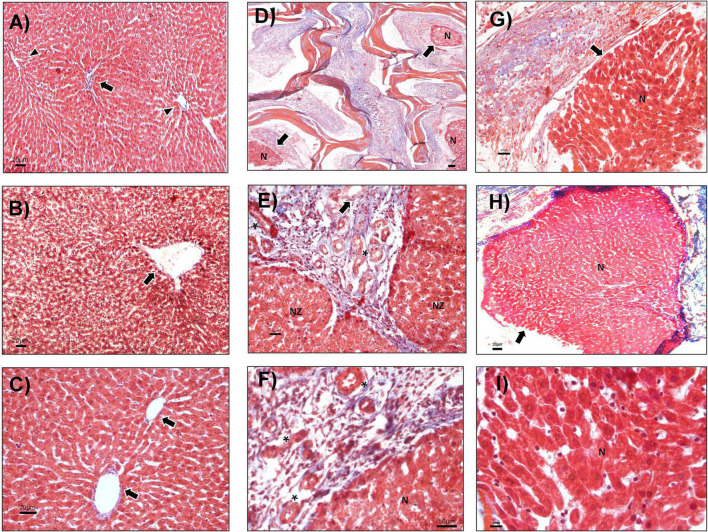
Nodules of the hepatic cell at CMS implantation day 21. **(A)** The liver without hepatectomy (sham), portal triads (arrow), and central vein (arrowheads), 40×. **(B)** PH sample, the central vein is shown (arrow), 100×. **(C)** Basal tissue from animals evaluated up to day 21, hepatocyte cords and two central veins (arrows), 100×. **(D–I)** PH + CMS implantation at day 21. **(D)** CMS zone (CZ) and cell nodules (arrows), 40×. **(E)** Boundaries between the NZ and the CMS with ductular reaction (arrow), vessels (asterisk) 100×. **(F)** Limits between the nodules (N) and CMS, vessels (asterisks), 400×. **(G,H)** Nodules of liver tissue (arrow) in the CMS, 10×. **(I)** Hepatocytes and Küpffer cells in nodules at high magnification, 400×. Images with Masson’s trichrome staining.

As at the previous time points, there were no morphologic alterations at day 21 in the sham group or in the basal fragment evaluated (Figure 5A). Hepatocyte cords were maintained, and typical portal space and central veins were observed in all groups (Figures 5A–C). The CZ presented abundant connective tissue, multiple bands of collagen, and angiogenesis (Figure 5D). The presence of nodules of liver tissue inside the CMS was also identified (Figure 5E). Incipient inflammation composed of lymphocytes was detected (Figure 5F) and may be participating in the rearrangement of liver parenchyma, given that no evidence of necrosis or histological rejection was found. The cell nodules showed well-defined hepatocytes and the clear presence of Küpffer cells in the hepatic sinusoids (Figures 5G–I).

### Regulation of Liver Function and Biochemical Parameters After Collagen Matrix Scaffold Implantation

To evaluate liver function after PH, with and without the CMS, several biochemical parameters were evaluated and compared with the sham group. The levels of ALB, BIL, CHOL, TG, GLU, UR ac, CR-S, BUN, ALP, ALT, and AST from the sham, PH, and PH + CMS groups were evaluated at each time point (Table 1).

**TABLE 1:** Biochemical parameters evaluated in the sham, PH, and PH + CMS groups on days 3, 14, and 21.

	ALB (g/dL)	DBIL (mg/dL)	TBIL (mg/dL)	CHOL (mg/dL)	TG (mg/dL)	GLU (mg/dL)	UR ac (mg/dL)	CR-S (mg/dL)	BUN (mg/dL)	ALP (IU/L)	AST (IU/L)	ALT (IU/L)
Sham, day 3	1.35 ± 0.07	0.12 ± 0.02	0.29 ± 0.06	35.00 ± 1.41	112.50 ± 3.54	182.00 ± 8.49	0.90 ± 0.00	0.35 ± 0.03	24.00 ± 1.41	165.00 ± 7.07	105.50 ± 0.71	56.00 ± 4.24
Sham, day 14	1.55 ± 0.35	0.11 ± 0.01	0.39 ± 0.16	38.00 ± 1.41	103.50 ± 12.02	191.00 ± 12.73	1.10 ± 0.14	0.37 ± 0.03	25.00 ± 1.41	146.00 ± 15.56	119.00 ± 22.63	57.50 ± 4.95
Sham, day 21	1.45 ± 0.21	0.11 ± 0.01	0.25 ± 0.07	37.50 ± 2.12	112.00 ± 21.21	203.00 ± 9.90	1.00 ± 0.14	0.43 ± 0.06	24.00 ± 1.41	167.50 ± 17.68	111.85 ± 16.76	53.00 ± 5.66
Partial hepatectomy, day 3	1.23 ± 0.04	0.08 ± 0.04	0.28 ± 0.04	39.00 ± 1.41	117.50 ± 3.54	224.50 ± 24.75	1.20 ± 0.00	0.41 ± 0.02	19.00 ± 1.41	200.00 ± 7.07	77.50 ± 3.54	40.00 ± 2.83
Partial hepatectomy, day 14	1.20 ± 0.11	0.09 ± 0.04	0.30 ± 0.00	36.00 ± 0.71	125.50 ± 21.21	234.50 ± 14.14	1.20 ± 0.35	0.43 ± 0.04	18.00 ± 0.71	195.00 ± 9.90	82.50 ± 2.83	47.00 ± 2.12
Partial hepatectomy, day 21	1.16 ± 0.14	0.08 ± 0.03	0.32 ± 0.16	40.00 ± 0.71	107.0 ± 8.49	240.50 ± 4.95	1.20 ± 0.07	0.45 ± 0.04	23.00 ± 0.71	185.00 ± 16.26	84.50 ± 1.77	49.00 ± 9.19
Partial hepatectomy + CMS, day 3	1.35 ± 0.07	0.11 ± 0.01	0.25 ± 0.07	39.00 ± 1.41	106.50 ± 33.23	187.50 ± 10.61	0.90 ± 0.00	0.39 ± 0.02	16.50 ± 0.71	250.50 ± 12.02	90.00 ± 1.41	67.75 ± 6.86
Partial hepatectomy + CMS, day 14	1.55 ± 0.07	0.10 ± 0.04	0.29 ± 0.04	42.00 ± 2.12	107 ± 21.21	187.50 ± 8.49	0.90 ± 0.35	0.38 ± 0.14	15.70 ± 1.41	258.0 ± 35.36	85.00 ± 1.77	69.75 ± 4.24
Partial hepatectomy + CMS, day 21	1.45 ± 0.14	0.12 ± 0.03	0.28 ± 0.07	38.00 ± 1.41	109 ± 3.54	187.50 ± 31.11	0.90 ± 0.14	0.37 ± 0.01	15.50 ± 1.41	265.0 ± 3.54	87.00 ± 6.36	65.75 ± 6.72

*Mean ± standard deviation (SD).*

The values of the parameters were normal in all the cases, in accordance with other reports. However, in the comparison of groups at each time point, the PH itself caused ALB decrease in a time-dependent manner, whereas the PH + CMS group promoted an increase of that analyte at days 14 and 21, compared with the evaluation at day 3 (Table 2). Moreover, the PH group also showed an increase in CHOL, in accordance with the time progression (3 < 14 < 21 days). In contrast, there were no statistical differences in the PH + CMS group (Table 2). ALP, AST, and ALT showed differences mainly between the initial (day 3) and final (day 21) time points (Table 2). The sham group showed no alterations in any of the parameters evaluated (Table 2).

**TABLE 2:** Intragroup comparison of the biochemical parameters.

	ALB (g/dL)	DBIL (mg/dL)	TBIL (mg/dL)	CHOL (mg/dL)	TG (mg/dL)	GLU (mg/dL)	UR ac (mg/dL)	CR-S (mg/dL)	BUN (mg/dL)	ALP (IU/L)	AST (IU/L)	ALT (IU/L)
**SHAM**												
3 VS 14	N/S	N/S	N/S	**0.05**	N/S	N/S	N/S	N/S	N/S	N/S	N/S	N/S
3 VS 21	N/S	N/S	N/S	N/S	N/S	**0.05**	N/S	N/S	N/S	N/S	N/S	N/S
14 VS 21	N/S	N/S	N/S	N/S	N/S	N/S	N/S	N/S	N/S	N/S	N/S	N/S
**PH**												
3 VS 14	**0.001**	N/S	N/S	**0.05**	N/S	N/S	N/S	**0.05**	N/S	N/S	**0.001**	**0.05**
3 VS 21	**0.05**	N/S	N/S	**0.001**	N/S	N/S	**0.05**	N/S	**0.001**	**0.001**	**0.001**	N/S
14 VS 21	**0.05**	N/S	N/S	**0.05**	N/S	N/S	N/S	N/S	**0.05**	N/S	N/S	N/S
**PH + CMS**												
3 VS 14	**0.001**	N/S	N/S	N/S	N/S	N/S	N/S	N/S	N/S	N/S	N/S	N/S
3 VS 21	**0.05**	N/S	N/S	**0.05**	N/S	**0.001**	N/S	N/S	**0.001**	**0.001**	**0.001**	**0.05**
14 VS 21	N/S	N/S	**0.05**	N/S	N/S	**0.05**	N/S	N/S	**0.05**	N/S	0.05	N/S

*Mean ± standard error. p < 0.05.*

*ALB, albumin, DBIL, direct bilirubin; TBIL, total bilirubin; CHOL, cholesterol; TG, triglycerides; GLU, glucose; UR ac, uric acid; CR-S, creatinine; BUN, blood urea nitrogen; ALP, alkaline phosphatase; ALT, alanine aminotransferase; AST, aspartate aminotransferase; PH, partial hepatectomy; and CMS, collagen matrix scaffold. The numbers in bold (e.g., 0.05, 0.001) highlight the p-values.*

On one hand, the multiple comparisons between groups revealed that GLU increased slightly in the PH group, compared with the sham group, at all the time points of the study (Table 3). Interestingly, GLU decreased in the PH + CMS group at days 14 and 21, compared with the sham group. ALP increased in the PH and PH + CMS groups on days 3, 14, and 21 vs. the sham group. On the other hand, AST decreased in the PH vs. the sham group at days 3 and 14, whereas ALT diminished in the PH group but increased in the PH + CMS group on day 3 vs. the sham animals (Table 3). Moreover, the ALP, AST, and ALT levels were higher in the PH + CMS group vs. the PH group on day 3, and this increase in ALP was maintained at days 14 and 21 in the PH + CMS group vs. the PH group (Table 3).

**TABLE 3:** Intergroup comparison of the biochemical parameters.

	ALB (g/dL)	DBIL (mg/dL)	TBIL (mg/dL)	CHOL (mg/dL)	TG (mg/dL)	GLU (mg/dL)	UR ac (mg/dL)	CR-S (mg/dL)	BUN (mg/dL)	ALP (IU/L)	AST (IU/L)	ALT (IU/L)
**PH vs SHAM**												
3 days	**0.05**	N/S	N/S	N/S	N/S	**0.001**	N/S	**0.05**	**0.05**	**0.05**	**0.001**	**0.05**
14 days	N/S	N/S	**0.05**	N/S	N/S	**0.05**	N/S	N/S	**0.05**	**0.05**	**0.05**	N/S
21 days	N/S	N/S	N/S	**0.05**	N/S	**0.05**	N/S	**0.05**	N/S	N/S	N/S	N/S
**PH + CMS vs SHAM**												
3 days	**0.05**	N/S	N/S	**0.05**	N/S	N/S	N/S	N/S	**0.001**	**0.001**	**0.001**	**0.05**
14 days	N/S	N/S	**0.05**	N/S	N/S	**0.05**	N/S	N/S	**0.05**	**0.001**	**0.05**	N/S
21 days	N/S	N/S	N/S	**0.05**	N/S	**0.05**	N/S	**0.05**	N/S	**0.05**	N/S	N/S
**PH VS PH + CMS**												
3 days	**0.05**	N/S	N/S	N/S	N/S	**0.05**	**0.05**	N/S	**0.05**	**0.001**	**0.001**	**0.001**
14 days	N/S	N/S	N/S	N/S	N/S	N/S	N/S	N/S	N/S	**0.05**	N/S	N/S
21 days	N/S	N/S	N/S	**0.05**	N/S	N/S	**0.05**	N/S	N/S	**0.05**	N/S	N/S

*Mean ± standard error. p < 0.05.*

*ALB, albumin, DBIL, direct bilirubin; TBIL, total bilirubin; CHOL, cholesterol; TG, triglycerides; GLU, glucose; UR ac, uric acid; CR-S, creatinine; BUN, blood urea nitrogen; ALP, alkaline phosphatase; ALT, alanine aminotransferase; AST, aspartate aminotransferase; PH, partial hepatectomy; and CMS, collagen matrix scaffold. The numbers in bold (e.g., 0.05, 0.001) highlight the p-values.*

## Discussion

The liver is an organ with a unique and extraordinary regeneration capacity, recovering its functions and restoring the resected volume (22, 23). However, its regeneration capacity can be impaired after repeated chronic injuries, causing excessive accumulation of ECM, which can ultimately lead to the development of cirrhosis or hepatocellular carcinoma (24). In advanced stages of damage, transplantation is the current therapeutic solution, for addressing this impairment.

Advances in liver regenerative medicine have attempted to use biomaterial scaffolds to simulate native conditions, providing a niche for the proliferation of parenchymal cells. Collagen has been considered an excellent protein in the design of synthetic and natural scaffolds (25–28). It displays low immunogenicity, is biocompatible and biodegradable, and regulates cellular processes, such as adhesion, migration, and differentiation (29).

Different strategies have been explored to obtain pure collagen to produce scaffolds. After extraction and purification, its recombinant form is obtained by incorporating techniques, such as lyophilization and electrospinning, to produce collagen scaffolds (30, 31). However, the application of physical or chemical treatments is needed to achieve intermolecular cross-linking of the collagen, promoting changes in the properties of the collagen protein (29, 31). CMS, obtained from the bovine condyle, is a natural biomaterial composed of 80% collagen (12). In this study, we evaluated the regenerative properties of CMS implantation in the rat liver. The model was developed with three principal purposes: (1) to evaluate the possibility of replacing the extirpated liver mass with the collagen scaffold, (2) to determinate the biocompatibility of the xenogenic implant in the liver, and (3) to explore the proliferation process of the liver in the presence of CMS.

The strategy most related to replacing the volume of an extirpated mass is organ decellularization. However, physical and chemical treatments to remove the allogeneic or xenogeneic cellular antigens are needed (32, 33). In our model, we found that it was possible to replace only a selected portion of the liver with the xenogenic CMS, reducing clinical and surgical complications, compared with the decellularization process. The animal model was successfully reproducible, confirming that the CMS can be tailored to the size and form required, as previously reported (13). Regarding biocompatibility and the regeneration process, we selected PH as it is the most common model for regeneration. Given that no dysregulated inflammation has been reported in this process, we incorporated the CMS into the PH model.

Serious blood flow alterations during regeneration have been described in PH models, as well as the induction of mediator production [e.g., NO and interleukin (IL)-6] that stimulates hepatic stellate cells (HSC) and consequently promotes the angiogenesis process (23, 34, 35). In our model, angiogenesis was observed starting at day 3 and increased at day 14. This result is in accordance with the timing of the angiogenic phase, which occurs around 72 h after PH (24).

Additionally, some authors suggest that a hypertrophic reaction and hepatocyte proliferation are the main events during hepatic regeneration after PH (36–38). Moreover, recent evidence has shown that a ductular reaction is also present during regeneration in PH, chiefly located close to the damaged area. Authors have also reported local inflammation in response to liver damage (39). In accordance with these reports, our animals that underwent PH displayed a ductular reaction at day 3 (data not shown). In the PH + CMS group, in addition to a ductular reaction, we found a transitory presence of inflammatory cells (neutrophils and lymphocytes) at day 3 and day 14 surrounding the CMS, respectively. However, both the ductular and inflammatory reactions were practically absent at day 21, but neoformation vessels were evident. Future studies are needed to evaluate the cytokine production during ductular reactions in the PH + CMS group.

To evaluate the proliferation in nodules, we performed immunolabeling with Ki-67 protein; however, we did not observe a positive label in the transitory zone, CZ, and native tissue (data not shown).

It has been reported that Ki-67 and BrdU dramatically decrease after 3 days of PH in rats (39–41). Moreover, thymidine-labeling studies have shown that hepatocytes in the remaining liver after PH divide once or twice to restore the number of hepatocytes at 3–4 days, with a maximum of proliferation at 24 h (42). Despite the fact that we also performed immunohistochemical analysis of Ki-67 on day 3, the label was not observed (data not shown). It is possible that in our study on day 3, a wave of proliferation was finished or paused. The evaluation of kinases and cyclins of the cell cycle could be crucial to evaluate the proliferation of hepatocytes and non-parenchyma cells in a near future. Despite Ki-67 being negative, our histological analysis showed evident cells (like hepatocytes) in the trabecula spaces at day 21 compared with days 3 and 14 in the PH + CMS group. In addition, it is important to add that it is possible that other processes could be involved in the negative phenotype of proliferation, which includes the size of PH, age of animals, and effect of xenoimplant (42–44). Moreover, in future studies to explore regeneration, we also considered using the most common experimental model of 70% hepatectomy in the presence of CMS and using several strategies to evaluate the proliferation process and other important molecular aspects (45).

Regarding the biocompatibility of CMS, we observed no areas of necrosis or extensive inflammatory reaction in the CZ or the limits close to the NZ. However, on day 14, some areas displayed FBGCs, including areas with non-absorbable sutures. The presence of FBGCs is usually considered a frustrating phagocytosis process for eliminating foreign material (46). The presence of FBGCs caused by sutures is a common feature in clinical procedures (47). Therefore, future studies using absorbable material are needed. In contrast, some studies suggest that the encapsulation of material by giant cells is then eliminated by macrophages (46). Nevertheless, our histological evidence showed no massive arrival of macrophages to eliminate the biomaterial, and FBGCs were not seen at day 21. Local mechanisms that include the activity of metalloproteinases may participate in the biosorption/enzymatic degradation of the biomaterial. Thus, further studies focusing on the degradation process of the CMS are required.

Regarding the presence of hepatocytes in the CMS at day 3, the histological evidence and the characteristics of the biomaterial (porous scaffold property) suggest that hepatocytes were carried off by the bloodstream. However, nodules derived from hepatocytes were clearly found inside the CMS at day 21. The histological evidence strongly suggests the intense presence of hepatic cells. Even so, it will be necessary to perform a study with evaluations at early (1–2 days) and longer than 21 days to assess regeneration events in our model and other percentages of PH. Some independent samples that were evaluated at 30 days postimplantation showed coalescence of the cell nodules (data not shown).

Since 1979, hepatic enzymes (ALP, AST, and ALT) have been defined as biomarkers for monitoring the structural integrity and damage of the liver (48, 49). It is currently known that different ratios of those enzymes, as well as their production, can be regulated differently according to the liver insult (49). In contrast, negative alterations or signs of hepatic dysfunction have been observed in clinical practice, in lobectomy or wedge resection models, and in PH animal models. Furthermore, the recovery of biochemical levels is reported to begin 3 days after the surgical process (15, 50, 51). In our study, different metabolites directed toward liver function were evaluated, and in general, the biochemical values were within the reference ranges reported for Wistar rats (14, 52). Our study revealed a reduction of ALB in the PH + CMS group. However, the comparative analysis of groups showed that the said decrease occurred only after 3 days in the PH group, correlating with previous reports (52). Altered GLU levels were also observed in the animals, and values were increased in the PH group vs. the sham group. GLU levels might be affected by a reduced number of hepatocytes, thereby making the liver inefficient for capturing GLU, with its blood levels tending to rise (15, 48). However, in the PH + CMS group, GLU levels were slightly increased. The elevation of ALP may be due to the surgical procedure and the stress of organ management, which causes damage to the hepatocytes and portal triads, promoting focal cholestasis but not generating hyperbilirubinemia (53). ALP is also associated with liver regeneration. Nevertheless, using the total serum level of ALP as a marker for liver regeneration remains controversial. ALP isoenzymes have been described to provide a better understanding of regenerative mechanisms, but the main information available is still related to total ALP (52). Studies of 10% hepatectomy show that ALP remains elevated 2 weeks after PH but then starts to decrease (15, 53). We observed the said increase, but it remained at day 21, perhaps due to the size of the hepatectomy and the elimination process resulting in the biosorption of the CMS. Identifying the molecular mechanisms involved in the degradation of this biomaterial is crucial.

The regulation of ALT and AST in human lobectomy and 70% hepatectomy in Wistar rats was increased after 3 postoperative days, but then registered values similar to those of the control (15, 50, 54). In contrast, we reported a decrease in ALT and AST in the PH and the PH + CMS groups on days 3 and 14. The normal level of ALT in rats is ∼52IU/L (52), which was the value in our control group. ALT and AST serum levels have been associated with short ischemic episodes but with no negative effect on regeneration capacity (55, 56).

## Conclusion

The xenoimplant of CMS in the liver of a rat displays biocompatibility, acquires the size and form required, and is bioabsorbable. It does not alter liver functions and allows the development of cell nodules that show the typical architecture of healthy liver parenchyma. This xenogenic material is a novel strategy that can reduce the challenges in the field of solid organ transplants, including liver transplantation, and can restore function in chronic liver diseases.

## Data Availability Statement

The raw data supporting the conclusions of this article will be made available by the authors, without undue reservation.

## Ethics Statement

The animal study was reviewed and approved by Ethics Committee of the School of Medicine at the Universidad Nacional Autónoma de México (UNAM). All procedures were performed according to official Mexican policy (SAGARPA, 1999). Our institution fulfills all technical specifications for the production, care, and use of laboratory animals and is certified by national law (NOM-062-ZOO-1999).

## Author Contributions

GG-R and CP-B contributed to the study design. MM-C, BL-M, JG-L, and JS-H contributed to the data collection. BL-M contributed to surgery and animal handling. GR-R, AA, AP-T, and DD-I contributed to pathological interpretation. ZM-A contributed to the biochemical evaluation. MM-C, GR-R, DD-I, and GG-R contributed to the manuscript writing. GG-R contributed to the critical revision of the manuscript. All authors read and approved the final manuscript.

## Conflict of Interest

The authors declare that the research was conducted in the absence of any commercial or financial relationships that could be construed as a potential conflict of interest.

## Publisher’s Note

All claims expressed in this article are solely those of the authors and do not necessarily represent those of their affiliated organizations, or those of the publisher, the editors and the reviewers. Any product that may be evaluated in this article, or claim that may be made by its manufacturer, is not guaranteed or endorsed by the publisher.

## Footnotes

1
www.graphpad.com
